# Organisational Support for Diabetes Nursing Teams, Self‐Assessed Knowledge, and Training Willingness: A Cross‐Sectional Study

**DOI:** 10.1002/nop2.70918

**Published:** 2026-10-15

**Authors:** Yanan Si, Jingfang Liu, Defang Zhang, Xiaohang Wang, Xiaohan Xu, Subo Liu

**Affiliations:** ^1^ Department of Endocrinology Shijiazhuang People's Hospital Shijiazhuang Hebei Province China; ^2^ Department of Burn and Plastic Surgery Shijiazhuang People's Hospital Shijiazhuang Hebei Province China; ^3^ Institute of Translational Medicine, Jiangsu Key Laboratory of Integrated Traditional Chinese and Western Medicine for Prevention and Treatment of Senile Diseases, Medical College Yangzhou University Yangzhou China; ^4^ Department of Clinical Sciences Liverpool School of Tropical Medicine Liverpool UK

## Abstract

**Aim:**

To evaluate nurses' perceptions of organisational support for a hospital‐wide diabetes nursing team system, examine differences across nursing roles and departments, and investigate the associations of perceived organisational support with nurses' self‐assessed diabetes knowledge and willingness to engage in further diabetes‐related training.

**Design:**

A cross‐sectional study.

**Methods:**

Registered nurses from endocrinology and non‐endocrinology departments across three sites of a tertiary hospital group in China were recruited through a hospital‐wide online survey. Organisational support was assessed using a structured instrument covering five domains of the diabetes nursing team system, together with measures of self‐assessed diabetes knowledge and willingness to engage in further training. A total of 337 nurses were included. Descriptive analyses, subgroup comparisons, correlation analyses and multivariable ordinal logistic regression were performed.

**Results:**

Nurses reported generally high levels of perceived organisational support for the diabetes nursing team system. Diabetes liaison nurses reported higher perceived training and competency support than non‐liaison nurses, whereas overall organisational support, self‐assessed knowledge and learning willingness were similar between endocrinology and non‐endocrinology nurses. Greater perceived training and competency support and professional development support were independently associated with stronger willingness to engage in further diabetes‐related training. Individual professional characteristics were not independently associated with learning willingness after adjustment.

**Conclusion:**

Perceived organisational support for training and professional development was associated with nurses' willingness to engage in further diabetes‐related training. Strengthening these organisational components may support continuing professional development within hospital‐wide diabetes nursing systems.

**Implications for the Profession and/or Patient Care:**

Nursing managers should consider accessible training, competency support and professional development opportunities as part of hospital‐wide strategies to strengthen nurses' capacity for diabetes care across specialist and non‐specialist departments.

**Impact:**

The findings highlight organisational conditions that may support continued learning within hospital‐wide diabetes nursing systems. They may inform nursing management strategies for workforce development in settings where diabetes care extends across multiple clinical departments.

**Reporting Method:**

The study is reported in accordance with the Strengthening the Reporting of Observational Studies in Epidemiology (STROBE) guidelines.

**Patient or Public Contribution:**

No patient or public contribution.

## 1 Introduction

Diabetes mellitus poses a substantial and growing burden on health systems worldwide, requiring sustained, high‐quality nursing care across the continuum of prevention, treatment and complication management (Chen et al. 2025; Care 2025). Health systems increasingly rely on nursing workforces to deliver complex chronic disease care, and nurses across multiple clinical departments are frequently involved in the monitoring, education and management of patients with diabetes. As diabetes prevalence increases and care pathways become more complex, the demand for well‐coordinated nursing teams with adequate training, role clarity and institutional support has intensified (Leon et al. 2025; Dorey et al. 2023; Olsson et al. 2025). This challenge extends beyond specialist diabetes services because patients with diabetes frequently receive care in medical, surgical, emergency, critical care and other clinical settings. Nurses working outside endocrinology therefore require sufficient diabetes‐related knowledge and access to appropriate clinical support to provide safe and consistent care (Committee ADAPP 2025; Ayman et al. 2023). Developing these capabilities across the nursing workforce has consequently become an important issue for nursing management and continuing professional development.

Specialist nursing models, including diabetes liaison nurses and multidisciplinary nursing teams, have been widely promoted to enhance the quality and consistency of diabetes care (Friel et al. 2022). Previous studies have demonstrated that specialist nursing involvement can improve glycaemic control, patient education and self‐management outcomes (Sun et al. 2025; Liang et al. 2025; Akiboye et al. 2021). However, the contribution of such models depends not only on the expertise of individual nurses but also on the organisational environment in which they work. Institutional arrangements that provide access to training, effective communication, clinical guidance, appropriate resources and opportunities for professional development can support nurses in developing and maintaining diabetes‐related capabilities (Aldahmashi et al. 2024). Conversely, limited training opportunities, unclear responsibilities, and uneven access to support may constrain the implementation of diabetes nursing systems, particularly in departments where diabetes care is not the primary clinical focus (Committee ADAPP 2025). This places greater emphasis on whether organisations provide the structures and resources needed to support nurses' diabetes‐related practice and continued professional development, rather than focusing solely on individual knowledge.

One organisational approach used to extend diabetes nursing support beyond specialist departments is the diabetes liaison nurse model. Within hospital‐wide diabetes nursing systems in China, diabetes liaison nurses are designated nurses based in individual clinical units who provide a link between specialist diabetes teams and nurses working in other departments (Song et al. 2025). Although this role shares some functions with diabetes specialist nursing, a diabetes liaison nurse is not necessarily equivalent to a diabetes specialist nurse. Internationally, similar coordinating functions may be undertaken by diabetes link nurses, ward‐based diabetes champions, or diabetes specialist nurses providing outreach and educational support, with the organisation, training and scope of these roles varying across clinical settings and healthcare systems (Martin et al. 2024; Gilligan et al. 2025). Liaison nurses generally retain their primary departmental responsibilities while assuming additional functions related to the implementation of diabetes care procedures, dissemination of training, clinical communication, patient education and quality improvement. This model is particularly relevant within hospital groups, which have increasingly been developed in China to integrate resources, standardise practice and strengthen workforce development across clinical departments and campuses (An et al. 2025). Because specialised diabetes competencies in China are often developed through hospital‐based training and continuing professional development, institutional training systems may have an important role in supporting nurses who provide diabetes care outside specialist settings (Wang et al. 2023; Fei et al. 2025). Evidence from Chinese hospitals nevertheless suggests that diabetes knowledge and training needs continue to vary among nurses working in non‐endocrinology departments (Song et al. 2025; Wang et al. 2023; Fei et al. 2025), highlighting the need to understand not only individual competency but also the organisational conditions under which such competency is developed and supported.

Assessing these organisational conditions requires a different perspective from simply measuring nurses' clinical knowledge or skills. Generic measures of perceived organisational support typically examine employees' broader perceptions of whether their organisation values their contribution, supports their work, and is concerned with their wellbeing (Eisenberger et al. 1986). In nursing research, perceived organisational support has been associated with professional self‐efficacy and career development (Ni et al. 2024). Such measures are useful for assessing employees' overall perceptions of organisational support, but they do not usually examine the specific structures through which support is delivered within a particular clinical service or nursing programme. In contrast, the present study focuses on concrete organisational components of a hospital‐wide diabetes nursing system, including training, communication, implementation support, resources and opportunities for professional development. Existing diabetes nursing studies have also commonly examined nurses' diabetes knowledge, training needs, or clinical competencies (Song et al. 2025; Wang et al. 2023; Fei et al. 2025), rather than organisational features such as the availability of training, communication pathways, implementation support, resources and professional development opportunities. The distinction is important because nurses' self‐assessed diabetes knowledge should not be interpreted as equivalent to objectively demonstrated knowledge. Previous evidence has shown that nurses' perceptions of their diabetes knowledge may not consistently correspond with performance on objective knowledge assessments (Clayton and Clayton 2024). Consequently, assessment of a diabetes nursing system should distinguish nurses' perceptions of their own knowledge from their perceptions of the organisational structures that support practice and continued learning. For this reason, the instrument used in the present study was designed as a context‐specific system assessment rather than as a general measure of perceived organisational support or an established measure of individual clinical competence. Such an approach may complement existing implementation research by providing information on how organisational systems are experienced by frontline nurses in routine practice (Green et al. 2024).

Despite the increasing emphasis on structured diabetes nursing teams and continuing professional development, evidence remains limited regarding how nurses across different departments and professional roles perceive organisational support for these systems and whether perceived support is associated with their self‐assessed knowledge and motivation for continued learning. Understanding these relationships may help nursing managers identify organisational features that are most relevant to workforce development rather than attributing differences in learning engagement solely to individual professional characteristics. Therefore, this study aimed to evaluate nurses' perceptions of organisational support for a hospital‐wide diabetes nursing team system, examine differences across nursing roles and departments, and investigate the associations of perceived organisational support with nurses' self‐assessed diabetes knowledge and willingness to engage in further diabetes‐related training.

## 2 Methods

### 2.1 Study Design and Setting

This study was designed as a cross‐sectional nursing management and health services research project to assess nurses' perceptions of the implementation and organisational support of a diabetes nursing team system within a hospital group. The study focused on organisational conditions relevant to nursing workforce development, including institutional training, coordination across clinical units, access to support and opportunities for professional development.

The study was conducted across three campuses of a tertiary hospital group in China. The hospital operates as an integrated system consisting of three geographically separate campuses, Jianhua, Fanxilu and Fangbei, under a unified governance structure, shared nursing management framework and coordinated clinical protocols. Within this organisational structure, hospital‐level nursing management systems coordinate training programmes, quality control processes and professional development initiatives across campuses, providing an institutional framework for the implementation of diabetes nursing team systems.

### 2.2 Operational Structure of the Diabetes Liaison Nurse System

Within the study setting, diabetes liaison nurses were designated nurses based in individual clinical units who supported the implementation of diabetes‐related nursing management within their departments. The broader function of this role is described in the Introduction, whereas its operational structure is reported here because liaison nurse status was included as a study variable. One diabetes liaison nurse was designated in each of 77 clinical departments and 10 emergency or outpatient units, resulting in 87 liaison nurses across the three campuses. In addition, the diabetes nursing team included eight core quality‐control members who supported hospital‐wide coordination, training, quality monitoring and implementation of diabetes nursing standards.

Liaison nurses remained based within their primary departments while undertaking additional responsibilities related to the implementation of diabetes‐related nursing procedures, dissemination of training content, health education, communication with the diabetes nursing team and supervision of relevant quality‐control requirements. To support these responsibilities, liaison nurses received additional structured training organised by the hospital nursing administration and diabetes nursing team. At a minimum, liaison nurses participated in diabetes‐related training once per quarter.

### 2.3 Development and Refinement of the Assessment Instrument

The assessment instrument was developed through an iterative and pragmatic process informed by established principles of questionnaire development, including literature‐informed item generation, expert review, pilot testing, item analysis and iterative refinement (Artino et al. 2014). This process was not based on a single formal psychometric development framework. Its purpose was to develop a context‐specific instrument for evaluating the diabetes nursing team system in routine nursing management practice rather than to establish a new general‐purpose psychometric scale.

The initial version of the instrument (V1) was developed based on a comprehensive review of international and Chinese literature on diabetes specialist nursing, nursing team management and hospital‐based training systems. This process was complemented by institutional policy review and expert consultations with senior nursing managers, diabetes specialist nurses and frontline clinical nurses.

Items were generated to reflect key components of diabetes nursing team implementation, including organisational structure and standardisation, training and competency support, communication and collaboration, resource allocation and incentives and organisational culture and professional development. These domains were defined conceptually to represent organisational components considered relevant to the hospital‐wide diabetes nursing system and were not specified a priori as a validated latent factor structure.

To enhance content relevance and clarity, the preliminary questionnaire was reviewed by a panel of five experts with experience in diabetes nursing management, nursing education and hospital quality improvement. Experts evaluated the items for relevance, clarity, and applicability to the hospital‐based diabetes nursing system. Based on their feedback, wording was revised, redundant items were removed, and the content was refined to improve alignment with the intended organisational domains.

Following expert review, the instrument underwent three rounds of pilot testing, referred to as V1, V2 and V3. The pilot samples comprised 62 nurses for V1, 31 nurses for V2 and 39 nurses for V3. Participants were recruited from all three hospital sites and represented different nurse levels, professional titles, levels of clinical experience and diabetes liaison nurse roles. Characteristics of participants in each pilot round are presented in File S1: Table S1. The pilot samples were used exclusively for instrument refinement and were not included in the final analytical sample of 337 nurses.

During each pilot phase, empirical data were used to examine item distributions, internal consistency, item‐total correlations and response patterns. These analyses were intended to identify redundancy, limited response variability and wording or feasibility problems rather than to provide formal psychometric validation. V1 was used primarily to identify redundancy and ceiling effects. V2 evaluated the streamlined structure and revised item wording, whereas V3 was used to confirm the feasibility and wording of the final version before implementation in the main study.

Based on the pilot findings and expert review, redundant or poorly performing items were removed, wording was refined to improve clarity and clinical relevance, and reverse‐scored items were introduced to reduce acquiescent responding. The instrument was progressively shortened to reduce respondent burden while maintaining coverage of the organisational components considered relevant to diabetes nursing team implementation.

The development, pilot testing and final application of the instrument are illustrated in Figure 1. Additional details regarding the three pilot samples, item refinement and changes between versions are provided in File S1 Part A and File S1: Table S2.

**FIGURE 1:**
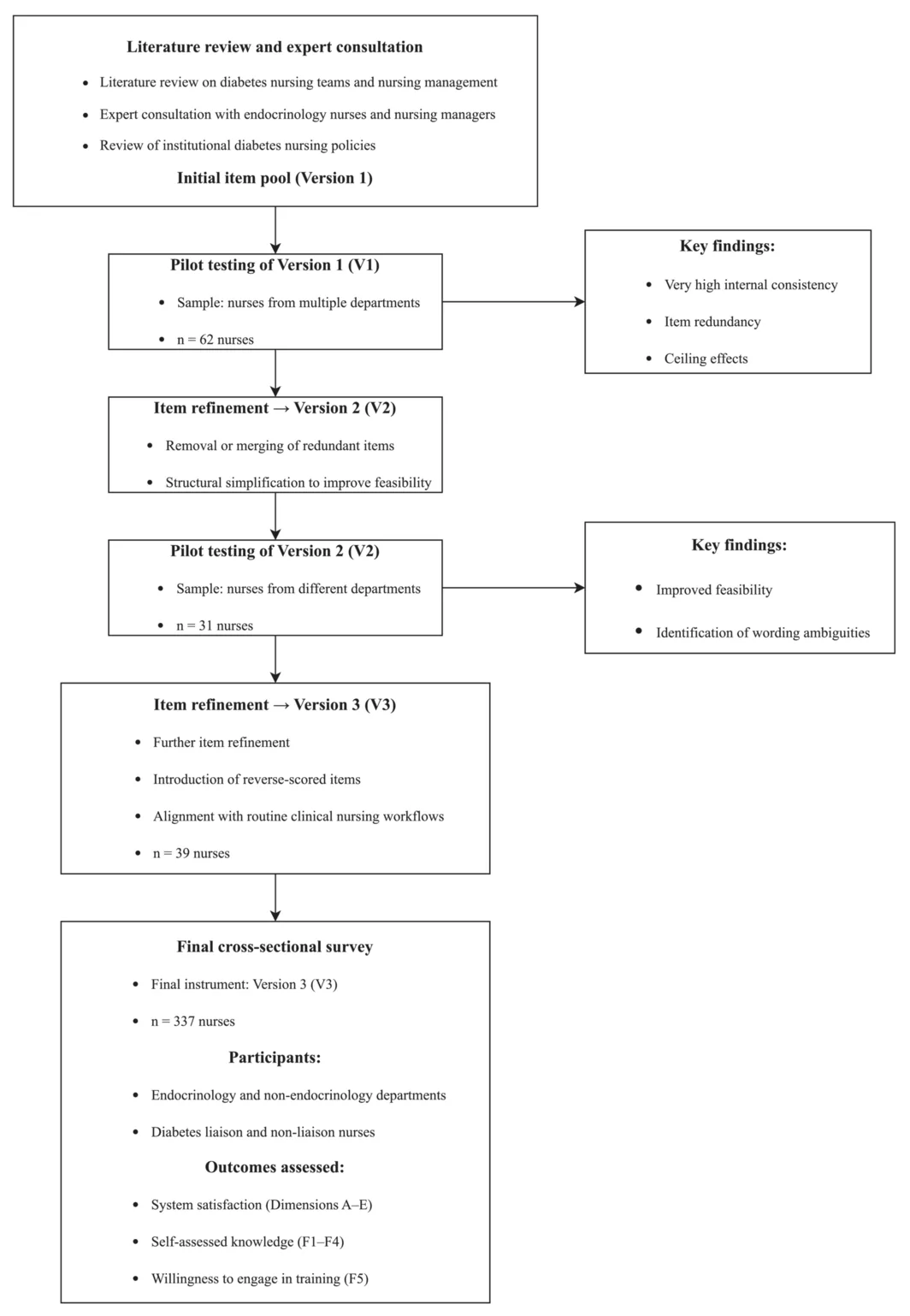
Development and implementation of the diabetes nursing team assessment system. The assessment instrument was developed through literature review, expert consultation and institutional policy review, followed by three rounds of pilot testing and iterative refinement (V1–V3). The final version was implemented in a cross‐sectional survey across endocrinology and non‐endocrinology departments to evaluate system‐level support for diabetes nursing teams and inform nursing management strategies. Pilot testing was conducted for iterative refinement and feasibility optimisation rather than formal psychometric validation.

The final instrument comprised four components with distinct measurement purposes, which were analysed separately as appropriate. The first component was the core system satisfaction scale, consisting of five conceptually defined organisational domains, referred to as dimensions A–E. Each dimension contained four items, giving a total of 20 items. The domains addressed system development and standardisation, training and competency support, communication and collaboration, resources and incentives and organisational culture and professional development. All items were rated on a 5‐point Likert scale from 1, strongly disagree, to 5, strongly agree. One negatively worded item was included in each dimension and reverse scored before calculation of dimension and total scores.

The second component consisted of four items assessing self‐assessed diabetes knowledge, F1–F4. These items measured nurses' perceptions of their capability in selected areas of diabetes care and were analysed separately from the organisational support dimensions. Their sum was used to calculate the self‐assessed diabetes knowledge score, F_total.

The third component was F5, a single item assessing willingness to engage in further systematic diabetes‐related training. F5 was treated as a separate outcome because it represented learning motivation rather than perceived knowledge and was not included in either F_total or the A–E system satisfaction score.

The fourth component was a 10‐item objective diabetes knowledge questionnaire, Q1–Q10. This questionnaire was retained as a supplementary descriptive measure and was not incorporated into the scoring of the organisational support scale, the self‐assessed knowledge score, or the primary inferential analyses. The present analysis did not formally examine agreement or correspondence between self‐assessed and objective diabetes knowledge.

The final instrument was applied primarily as a context‐specific nursing management assessment rather than as a formally validated psychometric scale. Measurement analyses in the main survey sample were therefore undertaken to characterise the performance of the instrument and to inform interpretation of its scores. File S1 Part B presents the internal consistency analyses and Exploratory Factor Analysis (EFA) performed using the final survey dataset. Cronbach's alpha was calculated for the overall system satisfaction scale and its individual domains. EFA was used to explore the structural characteristics of the 20‐item system satisfaction scale rather than to confirm the five conceptually defined domains. Sampling adequacy and factorability were assessed using the Kaiser–Meyer–Olkin measure and Bartlett's test of sphericity, and parallel analysis was used to inform the number of factors retained. The complete final survey instrument, Version 3, is provided in File S2.

### 2.4 Participants and Data Collection

Registered nurses from endocrinology and non‐endocrinology departments across the three hospital campuses were eligible to participate. Non‐endocrinology departments included internal medicine wards, surgical units, intensive care units, outpatient services and other clinical departments involved in diabetes care.

Eligible participants were registered nurses currently working in clinical departments across the hospital group and directly involved in patient care. Trainee nurses, student nurses and administrative nursing staff without direct patient care responsibilities were excluded.

Data were collected using an online survey platform. A questionnaire link was distributed hospital‐wide through internal communication channels to eligible nurses across departments and campuses. Participation was voluntary, and nurses accessed the questionnaire through the survey link. Because the survey link was openly distributed through hospital‐wide communication channels rather than through individualised invitations, the number of eligible nurses who received or viewed the invitation could not be reliably determined. A formal survey response rate could therefore not be calculated. All questionnaire items required a response before submission, thereby preventing item‐level missing data in submitted questionnaires.

Following completion of the V3 pilot phase and finalisation of the instrument, the main cross‐sectional survey was administered. A total of 337 questionnaires were submitted. All were complete and were included in the final analysis. There were therefore no partially completed questionnaires or item‐level missing data among submitted responses.

The final sample was also considered sufficient for the supplementary EFA. Methodological literature commonly suggests approximately 5–10 participants per item for EFA (Costello and Osborne 2005). The 20‐item core system satisfaction scale would therefore require approximately 100–200 participants under this rule of thumb. The final sample of 337 exceeded this threshold. Factorability was additionally assessed empirically using the Kaiser–Meyer–Olkin measure and Bartlett's test of sphericity.

### 2.5 Measures

Demographic and professional characteristics included hospital campus, department type, nursing level, professional title, years of clinical practice and diabetes liaison nurse status.

For the organisational support measures, scores for each of the five organisational domains were calculated by summing the four corresponding items after reverse scoring where required. The five domain scores were then summed to produce the overall system satisfaction score, Main_total, with higher scores indicating more favourable perceptions of organisational support for the diabetes nursing team system.

Negatively worded items A4, B4, C4, D4 and E4 were reverse scored as 6 minus the original response before score calculation. Self‐assessed diabetes knowledge, F_total, was calculated as the sum of F1–F4. Willingness to engage in further diabetes‐related training, F5, was analysed as a single‐item outcome.

### 2.6 Statistical Analysis

Descriptive statistics were used to summarise participant characteristics and study measures. Continuous variables were presented as mean and standard deviation or median and interquartile range, as appropriate, whereas categorical variables were presented as frequencies and percentages. Group comparisons were performed using independent‐samples *t*‐tests or Mann–Whitney *U* tests for two‐group comparisons and one‐way analysis of variance or Kruskal–Wallis tests for comparisons involving more than two groups. Associations between perceived organisational support, self‐assessed diabetes knowledge and willingness to engage in further training were examined using Spearman's rank correlation coefficients.

Because willingness to engage in further training was measured using a five‐category ordered response, multivariable ordinal logistic regression was used to examine factors independently associated with F5. Training and competency support and professional development support were included as the organisational predictors of primary interest. Years of clinical practice, professional title and diabetes liaison nurse status were included as professional characteristics. Adjusted odds ratios and 95% confidence intervals were estimated.

Prior to model construction, multicollinearity among independent variables was assessed using generalised variance inflation factors to evaluate the stability of the regression estimates.

Internal consistency of the final instrument was assessed using Cronbach's alpha. EFA was conducted as a supplementary analysis to explore the structural characteristics of the 20‐item system satisfaction scale. It was not used to confirm the five conceptually defined organisational domains or to establish formal construct validity. The number of factors retained was informed by parallel analysis.

All analyses were conducted using R software. A two‐sided *p* value < 0.05 was considered statistically significant. Detailed results of the supplementary reliability analyses, EFA and multicollinearity diagnostics are presented in File S1 Part B.

### 2.7 Ethical Considerations

The study was approved by the Hospital Research Ethics Committee of Shijiazhuang People's Hospital (Approval No. 2025(188)) on 27 November 2025. The study was conducted in accordance with the principles of the Declaration of Helsinki. Participation was voluntary and anonymous. Participants were informed of the purpose of the study before accessing the questionnaire, and informed consent was implied by completion and submission of the questionnaire. No directly identifiable personal information was collected.

## 3 Results

### 3.1 Participant Characteristics

A total of 337 nurses completed the survey and were included in the final analysis. Participants were recruited from three hospital campuses, with the majority from Jianhua (77.2%), followed by Fanxilu (16.9%) and Fangbei (5.9%). Nurses were drawn from both endocrinology (41.2%) and non‐endocrinology departments (58.8%). Nearly half of the participants were senior‐level nurses (N3–N4, 49.3%), and most held supervisory or senior professional titles. The mean duration of clinical practice was 13.0 ± 6.1 years. Of the 337 participants, 56 (16.6%) were diabetes liaison nurses. Detailed participant characteristics are presented in Table 1.

**TABLE 1:** Characteristics of the study participants (*n* = 337).

Variable	Category	*n* (%) or mean ± SD
Hospital site	Jianhua	260 (77.2)
	Fanxilu	57 (16.9)
	Fangbei	20 (5.9)
Department type	Endocrinology	139 (41.2)
	Non‐endocrinology	198 (58.8)
Nurse level	N0–N1	36 (10.7)
	N2	135 (40.1)
	N3–N4	166 (49.3)
Professional title	Nurse	14 (4.2)
	Senior nurse	73 (21.7)
	Supervisor nurse	239 (70.9)
	Chief nurse	11 (3.3)
Years of practice (years)	—	13.0 ± 6.1
Diabetes liaison nurse	Yes	56 (16.6)
	No	281 (83.4)

*Note:* Data are presented as number (percentage) or mean ± standard deviation.

### 3.2 Organisational Support, Self‐Assessed Knowledge and Learning Willingness

Nurses generally reported favourable perceptions of organisational support for the diabetes nursing team system. Mean scores across the five organisational domains ranged from 15.5 to 16.0 out of a possible 20, and the mean overall system satisfaction score was 79.1 ± 17.1 out of 100. The mean self‐assessed diabetes knowledge score was 16.9 ± 4.6 out of 20, whereas mean willingness to engage in further diabetes‐related training was 4.2 ± 1.2 out of 5. Detailed results are presented in Table 2.

**TABLE 2:** Overall scores of the diabetes nursing team system satisfaction scale.

Measure	Mean ± SD	Min–max
Dimension A: System construction and standardisation	16.0 ± 3.7	4–20
Dimension B: Training and competency support	15.9 ± 3.7	4–20
Dimension C: Communication and collaboration	16.0 ± 3.6	4–20
Dimension D: Resources and incentives	15.7 ± 3.7	4–20
Dimension E: Organisational culture and professional development	15.5 ± 3.5	4–20
Total score (A–E)	79.1 ± 17.1	24–100
Self‐assessed knowledge (F1–F4)	16.9 ± 4.6	4–20
Willingness to learn (F5)	4.2 ± 1.2	1–5

*Note:* Higher scores indicate greater perceived satisfaction or stronger learning willingness.

### 3.3 Measurement Properties of the Instrument

The 20‐item system satisfaction scale demonstrated high overall internal consistency in the final survey sample, with a Cronbach's α of 0.917. Cronbach's α values for the individual four‐item domains ranged from 0.54 to 0.61, indicating low to questionable internal consistency at the domain level. Accordingly, subsequent findings based on individual domain scores should be interpreted cautiously. Detailed reliability results are provided in File S1: Table S3.

EFA did not reproduce a clear structure corresponding to the five conceptually defined organisational domains. Although the data were suitable for factor analysis, with a Kaiser–Meyer–Olkin value of 0.965 and a significant Bartlett's test of sphericity (*χ* ^2^ = 9910.98, df = 190, *p* < 0.001), several items showed cross‐domain overlap and reverse‐worded items tended to cluster together. This pattern suggests that wording effects may have contributed to the observed structure and supports interpretation of the instrument as a context‐specific system assessment tool rather than an established psychometric scale. Full factor loadings and parallel analysis results are provided in File S1: Table S4 and Figure S1.

No problematic multicollinearity was identified among variables included in the multivariable model, with all adjusted GVIF values below 2. Detailed diagnostics are presented in File S1: Table S5.

### 3.4 Comparisons Across Nursing Roles and Departments

Perceptions of the diabetes nursing team system were broadly similar between nurses working in endocrinology and non‐endocrinology departments. No significant differences were observed in overall system satisfaction, the five organisational domains, self‐assessed diabetes knowledge, or willingness to engage in further training (all *p* > 0.05).

The main role‐based difference concerned training and competency support, with diabetes liaison nurses reporting higher Dimension B scores than non‐liaison nurses (*p* = 0.032). Professional title was also associated with perceived professional development support, with Dimension E scores differing across title groups (*p* = 0.012). Other comparisons according to liaison status and nurse level were largely non‐significant and are summarised in Tables 3 and 4.

**TABLE 3:** Comparison of scale scores across key nurse subgroups.

Measure	Endocrinology (*n* = 139)	Non‐endocrinology (*n* = 198)	*p*	Liaison nurse (*n* = 56)	Non‐liaison nurse (*n* = 281)	*p*
Dimension A	16.0 ± 3.6	16.0 ± 3.8	0.952	16.2 ± 3.5	15.9 ± 3.7	0.698
Dimension B	15.8 ± 3.5	15.9 ± 3.9	0.522	16.6 ± 3.8	15.7 ± 3.7	0.032
Dimension C	16.0 ± 3.5	16.0 ± 3.7	0.657	16.6 ± 3.8	15.9 ± 3.6	0.054
Dimension D	15.5 ± 3.8	15.8 ± 3.7	0.445	15.6 ± 3.8	15.7 ± 3.7	0.831
Dimension E	15.4 ± 3.5	15.6 ± 3.6	0.524	15.7 ± 3.5	15.5 ± 3.5	0.657
Total score (A–E)	78.8 ± 16.4	79.3 ± 17.6	0.623	80.6 ± 16.6	78.8 ± 17.2	0.340
Self‐assessed knowledge (F_total)	17.1 ± 4.4	16.8 ± 4.7	0.447	17.1 ± 4.7	16.9 ± 4.6	0.558
Willingness to learn (F5)	4.2 ± 1.2	4.2 ± 1.2	0.857	4.2 ± 1.3	4.2 ± 1.2	0.834

*Note:* Group comparisons were performed using independent‐samples *t*‐tests or Mann–Whitney *U* tests, as appropriate.

**TABLE 4:** Comparison of knowledge and learning willingness by (a) nurse level and (b) professional title.

(a)				
Measure	N0–N1 (*n* = 36)	N2 (*n* = 135)	N3–N4 (*n* = 166)	*p*
Self‐assessed knowledge (F_total)	16.4 ± 5.6	17.6 ± 3.7	16.4 ± 5	0.241
Willingness to learn (F5)	4.1 ± 1.4	4.3 ± 1	4.0 ± 1.3	0.166
Training support (Dimension B)	15.2 ± 4.2	16.3 ± 3.3	15.6 ± 4	0.366
Professional development (Dimension E)	15.6 ± 3.7	16.1 ± 3.1	15 ± 3.8	0.066

(b)					
Measure	Nurse (*n* = 14)	Senior nurse (*n* = 73)	Supervisor nurse (*n* = 239)	Chief nurse (*n* = 11)	*p*
Self‐assessed knowledge (F_total)	14.8 ± 6.4	17.9 ± 3.8	16.8 ± 4.6	15.5 ± 6.2	0.164
Willingness to learn (F5)	3.4 ± 1.7	4.4 ± 1	4.1 ± 1.2	3.9 ± 1.6	0.106
Training support (Dimension B)	13.9 ± 4.9	16.6 ± 3.3	15.8 ± 3.7	15.5 ± 5.1	0.155
Professional development (Dimension E)	14.9 ± 4.3	16.6 ± 3	15.3 ± 3.6	14.2 ± 3.6	0.012

*Note:* Group differences were assessed using one‐way ANOVA or Kruskal–Wallis tests.

### 3.5 Associations of Organisational Support With Self‐Assessed Knowledge and Learning Willingness

Overall system satisfaction was positively correlated with self‐assessed diabetes knowledge (*r* = 0.683, *p* < 0.001). Training and competency support showed a positive correlation with willingness to engage in further diabetes‐related training (*r* = 0.641, *p* < 0.001), as did perceived professional development support (*r* = 0.622, *p* < 0.001). Detailed correlation results are presented in Table 5.

**TABLE 5:** Correlations between system satisfaction, knowledge and learning willingness.

Variables	Correlation coefficient (*r*)	*p*
Total score (A–E) vs. self‐assessed knowledge (F_total)	0.683	< 0.001
Training support (B) vs. willingness to learn (F5)	0.641	< 0.001
Professional development (E) vs. willingness to learn (F5)	0.622	< 0.001

*Note:* Spearman's rank correlation coefficients were calculated.

### 3.6 Factors Associated With Willingness to Engage in Further Diabetes‐Related Training

In the multivariable ordinal logistic regression model, both training and competency support and professional development support remained independently associated with greater willingness to engage in further diabetes‐related training (Table 6). Each one‐point increase in training and competency support was associated with higher odds of reporting greater learning willingness (OR = 1.54, 95% CI: 1.36–1.76, *p* < 0.001). Higher professional development support was similarly associated with greater learning willingness (OR = 1.40, 95% CI: 1.22–1.60, *p* < 0.001).

**TABLE 6:** Multivariable analysis of factors associated with willingness to engage in further diabetes‐related training (*n* = 337).

Variable	Estimate (*β*/OR)	95% CI	*p*
System‐level factors			
Training and competency support (Dimension B)	1.54	1.36–1.76	< 0.001
Organisational culture and professional development (Dimension E)	1.40	1.22–1.60	< 0.001
Individual nurse characteristics			
Years of clinical practice (years)	1.01	0.95–1.06	0.844
Professional title (ref: nurse)			
Senior nurse	1.25	0.36–4.39	0.726
Supervisor nurse	1.37	0.38–4.97	0.633
Chief nurse	1.18	0.16–8.70	0.872
Diabetes liaison nurse (Yes vs. No)	0.64	0.33–1.24	0.183

*Note:* Values are presented as odds ratios (ORs) with 95% confidence intervals from ordinal logistic regression (proportional odds model). Willingness to engage in further training (F5) was measured on a 5‐point Likert scale, with higher scores indicating stronger learning willingness. System‐level factors were entered as continuous variables based on summed dimension scores. Professional title was included as a categorical variable, with ‘Nurse’ as the reference category.

By contrast, the individual professional characteristics included in the model were not independently associated with learning willingness after adjustment. Years of clinical practice (OR = 1.01, 95% CI: 0.95–1.06, *p* = 0.844) and diabetes liaison nurse status (OR = 0.64, 95% CI: 0.33–1.24, *p* = 0.183) were not significantly associated with the outcome, and no independent association was observed for professional title. Overall, the adjusted analysis showed that perceived organisational training support and professional development opportunities were more closely associated with learning willingness than the individual professional characteristics examined.

## 4 Discussion

The principal result of this study was that nurses' willingness to engage in further diabetes‐related training was more strongly associated with perceived training support and professional development opportunities than with the individual professional characteristics included in the multivariable model. Overall perceptions of the hospital‐wide diabetes nursing team system were favourable, and organisational support was also positively related to self‐assessed diabetes knowledge. Diabetes liaison nurses reported higher training and competency support than non‐liaison nurses, although liaison status itself was not independently associated with learning willingness after adjustment. This pattern places the organisational environment, rather than professional seniority or role designation alone, at the centre of the interpretation.

From an organisational support perspective, access to training, professional guidance, and opportunities for development can be understood as resources that shape how nurses engage with continuing learning. This interpretation is compatible with organisational support theory and the job demands–resources framework, both of which emphasise the role of workplace resources in supporting engagement and professional functioning (Aungsuroch et al. 2024; Xin et al. 2023). Organisational training structures and professional development opportunities may therefore contribute to an environment in which nurses feel better supported to maintain and extend their diabetes‐related capabilities (Aldahmashi et al. 2024; Kongkar et al. 2025). Similar associations between favourable work environments, perceived organisational support and professional engagement have been reported in nursing research (Kohnen et al. 2023; Mi and Xi 2025). The present study does not demonstrate that these organisational resources cause greater learning motivation, but it does suggest that nurses' perceptions of available support are closely linked to their willingness to continue developing their skills. Given the limited internal consistency of the individual organisational domains, however, the associations involving training support and professional development should be regarded as exploratory and require replication using independently validated measures.

A separate issue concerns the interpretation of self‐assessed diabetes knowledge. Although participants reported relatively high confidence in their diabetes‐related capabilities, self‐assessment should not be treated as equivalent to objectively demonstrated knowledge or clinical competence. Previous work has shown that nurses' perceived diabetes knowledge may not consistently correspond with scores on objective knowledge assessments (Clayton and Clayton 2024). This distinction is important because the positive association observed between organisational support and self‐assessed knowledge may partly reflect greater confidence, familiarity with available resources, or perceived preparedness rather than objectively verified competence. Supportive training environments may strengthen confidence and engagement, but high self‐assessed knowledge does not remove the need for continuing education or competency assessment.

The organisational context in which these results were observed also matters. International evidence indicates that nurses' diabetes knowledge differs across countries and healthcare settings, with variation potentially related to differences in nursing education, professional roles, continuing education systems, and the organisation of diabetes services (Alotaibi et al. 2016). The relatively high self‐assessed knowledge and favourable perceptions of support observed in this Chinese tertiary hospital group should therefore not be assumed to apply directly to other settings. At the same time, the absence of marked differences between endocrinology and non‐endocrinology nurses in the present study is noteworthy. Previous studies have reported stronger diabetes‐related competence or training engagement among nurses working in specialist settings (Song et al. 2025; Wang et al. 2023; Wu et al. 2021). One possible explanation is that the hospital‐wide training and coordination arrangements in this setting provided more comparable access to diabetes‐related support across departments. Because the study was cross‐sectional, however, this interpretation remains provisional and should not be taken as evidence that the organisational model eliminated departmental differences.

The liaison nurse comparison adds a further organisational perspective. Liaison nurses perceived greater training and competency support than nurses without this role, which is consistent with earlier work highlighting the value of specialist and coordinating roles in chronic disease management (Azzellino et al. 2025; Li et al. 2025; Lake et al. 2024). Yet liaison status was no longer associated with learning willingness once organisational support variables were included in the model. This weakens the interpretation that the role designation itself is the key mechanism. A more plausible explanation is that the role is accompanied by structured training, closer access to specialist support, clearer responsibilities and greater organisational recognition, all of which may shape how nurses experience opportunities for continued learning. Earlier literature has tended to focus on the clinical contributions of specialist or coordinating nurses (Li et al. 2025; Dailah 2024), whereas the present results point toward the organisational resources surrounding these roles as an additional area of interest. Variation in professional development scores across title groups also suggests that opportunities for advancement may not be experienced uniformly across the nursing workforce (Kaldal et al. 2024; Ni et al. 2025; Kurtović et al. 2024).

For nursing management, the practical implication is not simply to create additional specialist roles or isolated training sessions. Greater attention may be needed to the infrastructure that makes continuing learning possible across departments. Hospital‐level coordination of diabetes nursing, accessible training pathways, defined consultation routes and visible opportunities for professional development may help integrate diabetes‐related capability building into routine workforce management. Competency‐focused follow‐up may also be important, particularly because self‐perceived knowledge does not necessarily correspond to objectively assessed competence. For nursing leaders and educators, this suggests that continuing diabetes education may be more useful when embedded within an organisational system that provides ongoing access to guidance, opportunities for skill development, and support for applying learning in clinical practice. These principles may also be relevant to healthcare systems seeking to strengthen diabetes nursing capability beyond specialist endocrinology services, although the structure and responsibilities of liaison nursing roles are likely to vary across countries and healthcare settings. These organisational approaches remain hypotheses for implementation rather than demonstrated interventions, and prospective studies are needed to determine whether strengthening them improves nursing competence or patient outcomes.

Several limitations should be considered. The cross‐sectional design prevents causal interpretation of the observed associations. Nurses who are more motivated to learn may perceive organisational support more positively, just as stronger organisational support may encourage continued learning. Reliance on self‐reported measures also introduces the possibility of social desirability and common method bias. The single‐group setting further limits external validity. This hospital group has an integrated management structure, and the results may differ in institutions with different governance arrangements, staffing models, specialist roles, or continuing education systems. In addition, because the survey invitation was distributed through hospital‐wide communication channels without individualised invitations, a formal response rate could not be calculated. Voluntary participation through an online survey may also have introduced self‐selection bias. The use of self‐assessed diabetes knowledge introduces an additional interpretive limitation. Although an objective knowledge questionnaire was included as a supplementary descriptive component of the survey, the present analysis did not formally examine agreement between self‐assessed and objective knowledge. Consequently, the findings concerning self‐assessed knowledge should be interpreted as reflecting nurses' perceived capability rather than demonstrated diabetes knowledge or clinical competence. Future work could assess whether organisational support shows similar associations with objectively measured competence.

The assessment instrument itself also requires cautious interpretation. It was developed for this specific organisational context and should not be considered an established general measure of perceived organisational support. Although the overall 20‐item scale showed high internal consistency, the individual four‐item domains had Cronbach's α values ranging from 0.54 to 0.61, indicating low to questionable internal consistency. The EFA did not reproduce a clear structure corresponding to the five conceptually defined organisational domains, and the clustering of several reverse‐worded items suggests that wording effects may have influenced the factor pattern. For this reason, domain‐specific results should be interpreted conservatively, and the instrument is better regarded as a context‐specific pragmatic system assessment tool than as a fully validated psychometric scale. Independent validation, test–retest assessment, confirmatory factor analysis, measurement invariance testing and cross‐cultural evaluation would be required before broader use could be recommended.

Despite these limitations, the study extends the discussion of diabetes nursing development beyond individual knowledge and professional roles by considering the organisational conditions in which continuing learning occurs. The associations of training support and professional development with learning willingness identify potentially relevant targets for future longitudinal and implementation research. Such studies should determine whether strengthening these organisational resources leads to measurable improvements in nursing competence, care processes and patient outcomes across different healthcare settings.

## 5 Conclusion

Perceived training support and professional development opportunities were independently associated with nurses' willingness to engage in further diabetes‐related training, whereas the individual professional characteristics included in the model were not. Liaison nurses perceived greater training support, but liaison status itself did not remain independently associated with learning willingness after adjustment.

These results suggest that the organisational resources surrounding nursing roles may be more relevant to continued learning than role designation alone. Hospital‐wide training structures and accessible professional development pathways may therefore be useful areas for workforce development, although causal conclusions cannot be drawn from this cross‐sectional study. Further research in other organisational and cultural settings is needed to determine whether strengthening these forms of support improves objective nursing competence and patient care.

## Author Contributions

**Yanan Si:** conceptualization, methodology, investigation, writing – original draft, writing – review and editing. **Jingfang Liu:** investigation, writing – review and editing. **Defang Zhang:** investigation, writing – review and editing. **Xiaohang Wang:** writing – review and editing. **Xiaohan Xu:** writing – review and editing, formal analysis, software, supervision. **Subo Liu:** supervision, resources, writing – review and editing.

## Funding

This study was supported by the Hebei Provincial Health Commission (2024; Grant No. 20242106).

## Disclosure

The tool was not used to generate study data or perform statistical analyses. All scientific content, interpretations, analyses, conclusions, references, and final manuscript text were reviewed, verified and approved by the authors, who take full responsibility for the work. The AI tool was not listed as an author and did not assume responsibility for the manuscript.

## Ethics Statement

This study was approved by the Hospital Research Ethics Committee of Shijiazhuang People's Hospital (Approval No. 2025(188)) on 27 November 2025. The study was conducted in accordance with the principles of the Declaration of Helsinki. Participation was voluntary, and all data were collected anonymously. Informed consent to participate was implied by completion and submission of the questionnaire.

## Conflicts of Interest

The authors declare no conflicts of interest.

## Supporting information


**Table S1:** Characteristics of participants in the three pilot rounds.
**Table S2:** Summary of item refinement across pilot rounds (V1–V3).
**Table S3:** Internal consistency reliability of the instrument (Cronbach's alpha).
**Table S4:** Exploratory factor analysis loadings of the system satisfaction scale.
**Table S5:** Multicollinearity diagnostics for the multivariable model.
**Figure S1:** Parallel analysis for the main system satisfaction scale (Dimensions A–E).

## Data Availability

The data supporting the findings of this study are not publicly available because they are subject to institutional and ethical restrictions. Data may be available from the corresponding author upon reasonable request and subject to approval by the relevant institution and ethics committee.
