# Supplementary material for: Organisational Support for Diabetes Nursing Teams, Self‐Assessed Knowledge, and Training Willingness: A Cross‐Sectional Study
Source: Nurs Open. 2026 Oct 15;13(10):e70918. doi: 10.1002/nop2.70918 (PMC13673227; doi:10.1002/nop2.70918)
Supplement: Supplementary file 1 [file NOP2-13-e70918-s001.zip › nop270918-sup-0002-FileS1.docx]

**Final Survey Instrument (Version 3)**

**Diabetes Nursing Team System Satisfaction Survey**

**Instructions**

This survey aims to assess nurses’ perceptions of the current diabetes nursing team system and related management practices within the hospital group. Findings will be used for research on nursing management and may inform future service improvement strategies.

• Participation is voluntary and anonymous.

• No identifiable personal information is collected.

• Please answer based on your actual experience and perceptions.

• The survey takes approximately 5–8 minutes to complete.

• Responses are used solely for research purposes and will not affect individual evaluation or appraisal.

For each statement, please select one option:

1 = Strongly disagree 2 = Disagree 3 = Neutral 4 = Agree 5 = Strongly agree

This instrument is intended for system-level evaluation of diabetes nursing team implementation and training needs in routine clinical practice, rather than for formal psychometric validation.

**Section 1. Demographic and Professional Information**

1. Hospital campus

☐ Jianhua ☐ Fanxilu ☐ Fangbei

2. Department type

☐ Endocrinology

☐ Internal medicine (non-endocrinology)

☐ Surgery / Orthopaedics

☐ Operating room / Anaesthesia

☐ ICU / Emergency

☐ Outpatient clinic

☐ Obstetrics / Paediatrics

☐ Other: __________

3. Nursing level

☐ N0–N1 (Junior)

☐ N2 (Intermediate)

☐ N3–N4 (Senior)

4. Professional title

☐ Nurse ☐ Senior nurse ☐ Supervisor nurse ☐ Chief nurse

5. Years of clinical practice

____ years

6. Diabetes liaison nurse

☐ Yes ☐ No

**Section 2. Diabetes Nursing Team System Satisfaction Scale**

Dimension A. System development and standardisation (4 items)

A1. Clear and operational diabetes nursing policies or workflows (e.g. roles, quality control points, risk management procedures) are available and can be directly applied in daily practice.

A2. Key diabetes nursing processes (e.g. education, monitoring, insulin-related care) are largely consistent across hospital campuses, facilitating cross-campus collaboration.

A3. When deviations from diabetes care protocols occur, feedback and improvement mechanisms (e.g. review, feedback, retraining) are implemented rather than remaining at the document level.

A4*. In diabetes nursing practice, I often rely on personal experience because clear or easily accessible institutional procedures are lacking.

*Reverse-scored item

Dimension B. Training and competency support (4 items)

B1. The hospital or nursing administration provides ongoing and structured diabetes-related training for nurses outside endocrinology departments.

B2. Training content is tailored to the practical scenarios of my department (e.g. perioperative care, infection, geriatrics, pregnancy) and is directly applicable to my work.

B3. After training, follow-up mechanisms (e.g. assessment, mentorship, on-site supervision) help translate knowledge into daily practice.

B4*. When encountering diabetes-related problems, it is often unclear whom to contact for support, or timely guidance is difficult to obtain.

*Reverse-scored item

Dimension C. Communication and collaboration mechanisms (4 items)

C1. Clear communication channels for diabetes nursing collaboration (e.g. liaison nurses, messaging groups, consultation pathways) are available and easy to access.

C2. When patients experience glycaemic abnormalities or insulin-related issues, cross-disciplinary communication enables timely and coordinated decision-making.

C3. Diabetes-related nursing information is clearly handed over during patient transfers, discharge, or follow-up, without interruption.

C4*. In practice, diabetes-related collaboration is often delayed or overlooked due to high communication costs or complex procedures.

*Reverse-scored item

Dimension D. Resources and incentives (4 items)

D1. Essential resources for diabetes nursing (e.g. glucose monitoring supplies, educational materials, documentation tools) are adequately available.

D2. Work schedules allow sufficient time for key diabetes nursing activities (e.g. education, monitoring, insulin guidance).

D3. Diabetes nursing work is recognised in performance evaluation, appraisal, or departmental acknowledgment.

D4*. Diabetes nursing is often perceived as an additional burden, with insufficient resources, time, or incentives.

*Reverse-scored item

Dimension E. Organisational culture and professional development (4 items)

E1. The department or campus recognises the value of diabetes nursing (e.g. risk prevention, complication reduction, self-management support) and encourages nurse participation.

E2. My department actively promotes diabetes care quality improvement rather than focusing solely on task completion.

E3. Opportunities for professional development in diabetes nursing (e.g. advanced training, certification, quality projects) are available.

E4*. Long-term professional development or recognition in diabetes nursing is difficult to achieve in my department or hospital.

*Reverse-scored item

**Section 3. Self-assessed Diabetes Knowledge**

Knowledge self-assessment (F1–F4)

F1. I can recognise early signs of hypoglycaemia and provide appropriate management and education for conscious patients.

F2. I can guide patients on insulin-related procedures (e.g. injection site rotation, use of premixed insulin, troubleshooting).

F3. I can assess common issues related to blood glucose monitoring or CGM and determine when escalation is needed.

F4. I can provide targeted education on diet, exercise, and foot care and respond to common patient questions.

Willingness to engage in further training (F5)

F5. I am willing to participate in further systematic diabetes-related training if opportunities and resources are available.

Note: F5 reflects learning motivation and training needs and is analysed separately. It is not included in the knowledge self-assessment score.

**Scoring**

• All items are rated on a 5-point Likert scale (1–5).

• Reverse-scored items (A4, B4, C4, D4, E4) are scored as 6 − original score.

System satisfaction score (Main_total):

Sum of A1–A4, B1–B4, C1–C4, D1–D4, and E1–E4 after reverse scoring.

Theoretical range: 20–100, with higher scores indicating greater perceived system satisfaction.

Self-assessed knowledge score (F_total):

Sum of F1–F4 (range: 4–20).

Learning willingness (F5):

Analysed as a single-item outcome (mean, distribution, and associations).

**Appendix: Objective Diabetes Knowledge Test**

**Instructions**

The objective diabetes knowledge test consisted of 10 single-choice questions. Participants were instructed to select the single best answer for each question.

Each correct response was assigned 1 point, and incorrect responses were assigned 0 points. The total score therefore ranged from 0 to 10, with higher scores indicating greater objectively assessed diabetes-related knowledge.

The test was included as a supplementary descriptive component of the survey. It was not incorporated into the organisational support scale or the self-assessed diabetes knowledge score and was not included in the primary inferential analyses.

Acute glycaemic abnormalities and risk recognition

Q1. A patient receiving insulin treatment suddenly develops sweating, palpitations, and hand tremor but remains conscious. What is the most likely explanation?

A. Hyperglycaemic reaction

B. Early manifestations of hypoglycaemia

C. Diabetic ketoacidosis

D. Anxiety reaction

Q2. A patient with diabetes develops polydipsia, polyuria, fatigue, and deep, rapid breathing following an infection. Which condition should be considered first?

A. Uncomplicated hyperglycaemia

B. Hypoglycaemia

C. Diabetic ketoacidosis

D. Adverse drug reaction

Q3. Which patient is more likely to experience atypical symptoms during hypoglycaemia and therefore require closer glucose monitoring?

A. A young patient with newly diagnosed type 2 diabetes

B. An older patient with long-standing diabetes and peripheral neuropathy

C. A patient managed by dietary modification alone

D. A patient with obesity and chronically elevated blood glucose

Insulin administration and device-related problems

Q4. Before injecting a suspension insulin preparation, which step is most important?

A. Removing air from the device immediately before injection

B. Gently mixing the insulin until it appears uniformly milky

C. Massaging the injection site immediately after administration

D. Repeatedly using the same injection site to minimise pain

Q5. What is the main purpose of regularly rotating insulin injection sites?

A. To reduce injection-related pain

B. To prevent local infection

C. To prevent lipohypertrophy or lipoatrophy and maintain consistent insulin absorption

D. To make injection sites easier for patients to remember

Q6. A patient using an insulin pump has persistent blood glucose levels above 16 mmol/L. What should be checked first?

A. Whether the basal insulin rate should be immediately increased

B. Whether the patient has consumed excessive food

C. Whether the infusion set is blocked or disconnected

D. Whether a different type of insulin should be used

Blood glucose monitoring and quality control

Q7. Which situation is most likely to cause a falsely elevated capillary blood glucose reading?

A. Measuring before the disinfectant on the finger has completely dried

B. Measuring when sugar-containing substances remain on the fingers

C. Measuring in a low-temperature environment

D. Using a slightly insufficient blood sample

Q8. Which practice is most helpful for ensuring the accuracy of blood glucose meter measurements?

A. Repeatedly measuring blood glucose from the same finger

B. Comparing results obtained using test strips from different manufacturers

C. Performing regular quality-control testing using control solution

D. Recording the lowest value obtained from repeated measurements

Foot care and diabetes health education

Q9. Which finding most strongly indicates that a patient is at high risk of developing diabetic foot complications?

A. Dry skin of the feet

B. Occasional callus formation

C. Reduced peripheral sensory function

D. Mild lower-limb oedema

Q10. When providing health education to an older adult with diabetes, which issue should receive particular emphasis?

A. Maintaining blood glucose strictly within the normal range for people without diabetes

B. Avoiding hypoglycaemia and using individualised glycaemic targets

C. Performing high-intensity exercise regularly

D. Completely restricting carbohydrate intake

Answer Key and Scoring

Q1 = B; Q2 = C; Q3 = B; Q4 = B; Q5 = C; Q6 = C; Q7 = B; Q8 = C; Q9 = C; Q10 = B.

Each correct answer receives 1 point. The total objective diabetes knowledge score ranges from 0 to 10.

The objective knowledge score was retained as a supplementary descriptive measure. No formal analysis of agreement, correlation, or concordance between the objective knowledge score and F_total was undertaken in the present study.

**Version information**

Developed by the Diabetes Nursing Team, a tertiary hospital group in China.

Version: **V3.0**

Date: **22 December 2025**
