# Supplementary material for: Organisational Support for Diabetes Nursing Teams, Self‐Assessed Knowledge, and Training Willingness: A Cross‐Sectional Study
Source: Nurs Open. 2026 Oct 15;13(10):e70918. doi: 10.1002/nop2.70918 (PMC13673227; doi:10.1002/nop2.70918)
Supplement: Supplementary file 1 [file NOP2-13-e70918-s001.zip › nop270918-sup-0001-Supinfo.docx]

**Table S1. Characteristics of participants in the three pilot rounds**

| **Variable** | **Category** | **V1 (n = 62)** | **V2 (n = 31)** | **V3 (n = 39)** |
| --- | --- | --- | --- | --- |
| Hospital site | Jianhua | 42 (67.7) | 16 (51.6) | 21 (53.8) |
|  | Fanxilu | 15 (24.2) | 8 (25.8) | 16 (41.0) |
|  | Fangbei | 5 (8.1) | 7 (22.6) | 2 (5.1) |
| Nurse level | N0–N1 | 4 (6.5) | 4 (12.9) | 3 (7.7) |
|  | N2 | 27 (43.5) | 6 (19.4) | 15 (38.5) |
|  | N3–N4 | 31 (50.0) | 21 (67.7) | 21 (53.8) |
| Professional title | Nurse | 2 (3.2) | 1 (3.2) | 0 (0.0) |
|  | Senior nurse | 8 (12.9) | 4 (12.9) | 10 (25.6) |
|  | Supervisor nurse | 50 (80.6) | 24 (77.4) | 28 (71.8) |
|  | Chief nurse | 2 (3.2) | 2 (6.5) | 1 (2.6) |
| Years of practice (years) | Mean ± SD | 13.5 ± 4.9 | 15.1 ± 5.9 | 13.6 ± 6.4 |
| Diabetes liaison nurse | Yes | 56 (90.3) | 27 (87.1) | 21 (53.8) |
|  | No | 6 (9.7) | 4 (12.9) | 18 (46.2) |

Data are presented as number (percentage) or mean ± standard deviation. V1, V2, and V3 represent the three successive pilot rounds conducted during instrument refinement.

**Part A. Development and pilot testing of the assessment instrument (V1–V3)**

**Overview**

The assessment instrument used in the present study was developed to support the evaluation of a diabetes nursing team system from a nursing management and health services perspective. Instrument development followed an iterative, data-informed process incorporating literature review, expert consultation, and three rounds of pilot testing (V1–V3). The primary aim of the pilot phases was not formal scale validation, but pragmatic refinement of item content, structure, and feasibility to ensure relevance to routine nursing management practice. Accordingly, the pilot testing phases were not designed or reported as formal psychometric validation, but were used to support evidence-informed optimisation prior to large-scale implementation.

**Version 1 (V1): Initial framework development**

The initial version of the instrument (V1) was constructed based on a review of published literature on diabetes specialist nursing, nursing team organisation, training systems, and hospital-based diabetes care models, combined with expert consultations involving nursing managers and diabetes specialist nurses. V1 comprised six dimensions: system construction and standardisation (A), training and competency support (B), communication and collaboration (C), resources and incentives (D), professional identity and development (E), and self-assessed diabetes knowledge (F). Each dimension contained six items (36 items in total), alongside a 20-item objective diabetes knowledge checklist.

Pilot testing of V1 was conducted among 62 nurses. Descriptive analyses indicated consistently high mean scores across all dimensions, with most item means exceeding 4.0 on a 5-point Likert scale. Internal consistency was extremely high for all dimensions (Cronbach’s α ranging from 0.97 to approximately 1.00), and item–total correlations were uniformly strong. While these findings suggested internal coherence, they also indicated substantial item redundancy and pronounced ceiling effects, limiting the instrument’s ability to discriminate between respondents.

**Version 2 (V2): Structure simplification and content refinement**

Based on the V1 results, the instrument was revised to reduce redundancy and improve discriminative capacity. Dimensions A–E were each reduced from six to four items, yielding a 20-item core system satisfaction scale. To address acquiescence bias and improve response variability, one reverse-scored item was introduced in each of the A–E dimensions.

Dimension F (self-assessed diabetes knowledge) was refined to four core competency items (F1–F4), and a separate item assessing willingness to engage in further diabetes-related training (F5) was added as an independent indicator rather than part of the knowledge score. The objective knowledge checklist was shortened from 20 to 15 items, with items showing extremely high correct response rates or negative discrimination earmarked for revision.

Pilot testing of V2 was conducted among 31 nurses. Compared with V1, score distributions demonstrated increased variability. The overall internal consistency of the core system satisfaction scale (A–E) remained acceptable (α = 0.87), while individual dimensions showed lower α values, reflecting reduced redundancy and greater heterogeneity of responses. Item-level analyses identified several items with low item–total correlations, which informed targeted wording refinements rather than further item deletion.

**Version 3 (V3): Item wording optimisation and feasibility confirmation**

Version 3 focused on optimising item wording, enhancing contextual specificity, and improving feasibility for routine use. Items with low item–total correlations in V2 were revised to incorporate clearer management or clinical scenarios, while the overall structure (five system dimensions with four items each) was retained. Reverse-scored items were preserved to maintain response balance. The objective knowledge checklist was further reduced to 10 items, prioritising clinically relevant scenarios and removing items with persistent ceiling effects.

Pilot testing of V3 was conducted among 39 nurses. The core system satisfaction scale showed an overall Cronbach’s α of 0.94, while dimension-level α values ranged from 0.67 to 0.78. These reliability estimates were used only as preliminary indicators during instrument refinement and were not considered evidence of stable psychometric performance, particularly given the small pilot sample and the context-specific, pragmatic purpose of the instrument. Item distributions showed improved variability compared with earlier versions, and no further major problems affecting wording or feasibility were identified. V3 was therefore adopted for the final cross-sectional survey. Its measurement properties were subsequently evaluated in the larger final survey sample and are reported separately in Part B.

**Table S2. Summary of item refinement across pilot rounds (V1–V3)**

| **Dimension** | **Items in V1** | **Key findings from pilot testing** | **Rationale for modification** | **Items retained in V3** |
| --- | --- | --- | --- | --- |
| A. System construction and standardisation | 6 | Extremely high internal consistency (α≈0.99); strong item redundancy | Reduce redundancy; enhance discrimination | 4 |
| B. Training and competency support | 6 | High ceiling effects; overlapping training content | Streamline items; add reverse-scored item | 4 |
| C. Communication and collaboration | 6 | Near-identical item responses; limited variability | Merge similar concepts; add contextual specificity | 4 |
| D. Resources and incentives | 6 | High satisfaction scores; weaker discrimination in incentive items | Refine wording; focus on transparency and accessibility | 4 |
| E. Professional identity and development | 6 | Very high scores; limited variability | Reduce item number; introduce reverse-scored item | 4 |
| F. Self-assessed knowledge | 6 | High internal consistency; weak association with objective knowledge | Retain core competencies; separate learning willingness | 4 (+1 F5) |
| Objective knowledge checklist (Q) | 20 | High correct response rates; low discrimination | Remove/rewrite overly easy items; enhance clinical scenarios | 10 |

The iterative refinement process prioritised practical applicability, clarity, and feasibility for nursing management evaluation. Detailed psychometric validation was not the primary objective; rather, pilot testing results were used to inform evidence-based optimisation of item content and structure prior to large-scale implementation.

Version 2 (V2) functioned as an intermediate working version primarily used to identify item redundancy, ceiling effects, and wording issues. Rather than presenting V2 as a separate column, key findings from V2 pilot testing were incorporated into the rationale for item retention, deletion, and revision summarised in Table S2, which focuses on the transition from the initial framework (V1) to the final instrument (V3).

**Part B. Measurement properties of the assessment instrument in the final survey sample**

**Supplementary Figure S1. Parallel analysis for the main system satisfaction scale (dimensions A–E).**

**Table S3. Internal consistency reliability of the instrument (Cronbach’s alpha)**

| **Scale** | **Items** | **Cronbach_alpha** |
| --- | --- | --- |
| A_total | 4 | 0.594 |
| B_total | 4 | 0.610 |
| C_total | 4 | 0.563 |
| D_total | 4 | 0.613 |
| E_total | 4 | 0.540 |
| F_total | 4 | 0.981 |
| Main_total | 20 | 0.917 |

**Table S4. Exploratory factor analysis loadings of the system satisfaction scale**

| **Item** | **ML1** | **ML2** | **ML3** | **ML4** | **ML5** |
| --- | --- | --- | --- | --- | --- |
| A1 | 0.964 | 0.035 | -0.045 | -0.026 | -0.074 |
| A2 | 0.899 | 0.006 | 0.016 | -0.083 | -0.007 |
| A3 | 0.967 | -0.004 | -0.014 | -0.019 | -0.021 |
| A4 | -0.031 | 0.744 | -0.035 | 0.133 | 0.064 |
| B1 | 0.926 | -0.001 | 0.001 | 0.065 | -0.024 |
| B2 | 0.949 | -0.009 | 0.026 | 0.013 | 0.012 |
| B3 | 0.957 | -0.015 | -0.001 | 0.033 | 0.019 |
| B4 | 0.104 | 0.842 | -0.097 | -0.047 | -0.018 |
| C1 | 0.948 | 0.005 | -0.104 | 0.040 | 0.212 |
| C2 | 0.950 | -0.009 | -0.086 | 0.003 | 0.286 |
| C3 | 0.888 | -0.014 | 0.034 | 0.014 | 0.137 |
| C4 | 0.035 | 0.900 | -0.073 | 0.107 | -0.068 |
| D1 | 0.927 | 0.012 | 0.037 | 0.010 | 0.067 |
| D2 | 0.757 | -0.028 | 0.263 | 0.036 | 0.043 |
| D3 | 0.607 | -0.013 | 0.521 | 0.040 | -0.066 |
| D4 | 0.013 | 0.850 | 0.156 | -0.161 | 0.047 |
| E1 | 0.846 | 0.018 | 0.103 | 0.237 | -0.040 |
| E2 | 0.873 | 0.026 | 0.039 | 0.284 | -0.004 |
| E3 | 0.787 | -0.016 | 0.045 | 0.320 | 0.020 |
| E4 | -0.093 | 0.636 | 0.040 | -0.039 | -0.027 |

Factor loadings ≥0.40 are typically considered meaningful.

**Table S5. Multicollinearity diagnostics for the multivariable model**

| **Variable** | **GVIF** | **Df** | **GVIF_adj** |
| --- | --- | --- | --- |
| B_total | 3.198 | 1 | 1.788 |
| E_total | 3.201 | 1 | 1.789 |
| Work Years | 1.725 | 1 | 1.313 |
| Title | 1.836 | 3 | 1.107 |
| DM_liaison | 1.037 | 1 | 1.019 |
